# Multi-scale image edge detection based on spatial-frequency domain interactive attention

**DOI:** 10.3389/fnbot.2025.1550939

**Published:** 2025-04-28

**Authors:** Yongfei Guo, Bo Li, Wenyue Zhang, Weilong Dong

**Affiliations:** Xi'an Jieda Measurement & Control Co., Ltd., Xi'an, China

**Keywords:** spatial domain, frequency domain, multiple scale, interactive attention, edge detection

## Abstract

Due to the many difficulties in accurately locating edges or boundaries in images of animals, plants, buildings, and the like with complex backgrounds, edge detection has become one of the most challenging tasks in the field of computer vision and is also a key step in many computer vision applications. Although existing deep learning-based methods can detect the edges of images relatively well, when the image background is rather complex and the key target is small, accurately detecting the edge of the main body and removing background interference remains a daunting task. Therefore, this paper proposes a multi-scale edge detection network based on spatial-frequency domain interactive attention, aiming to achieve accurate detection of the edge of the main target on multiple scales. The use of the spatial-frequency domain interactive attention module can not only perform significant edge extraction by filtering out some interference in the frequency domain. Moreover, by utilizing the interaction between the frequency domain and the spatial domain, edge features at different scales can be extracted and analyzed more accurately. The obtained results are superior to the current edge detection networks in terms of performance indicators and output image quality.

## 1 Introduction

The edge information of images plays an important role in image understanding and analysis (Zhang and Shui, 2015; Jing et al., 2022a). In natural images, due to various complex situations such as large background interference and small main targets, accurately obtaining edges from images is undoubtedly a huge challenge in computer vision tasks (Romani et al., 2019; Shui and Zhang, 2012). With the continuous development of deep learning technology, it has shown strong potential and advantages in handling tasks related to image edge extraction.

Edge detection refers to an operation that extracts the contours of different objects and automatically ignores other details (Jing et al., 2022b; Zhang et al., 2017; Zhang and Sun, 2020). It can often provide important information in other image tasks, such as object detection (Amit et al., 2021), corner detection (Zhang and Shui, 2015; Zhang W. et al., 2023), image segmentation (Ghandorh et al., 2022), object tracking (Zhang and Sun, 2019), and image reconstruction (Li et al., 2023). These visual tasks all need to extract the boundaries of objects or perceive obvious edges from the original image. Therefore, only by stably detecting the details of object edges in an image can practical tasks be effectively completed. Traditional edge detection algorithms such as edge detection methods based on active contours (Cai et al., 2018; Ma et al., 2024) and edge detection methods based on multi-scale structures (Cui et al., 2021; Li et al., 2019; Zhang et al., 2019, 2014). Traditional edge detection methods rely on mathematical methods. When designing these methods, the attribute information of edges in the image needs to be considered and then corresponding mathematical methods are further used for processing in order to obtain the edge information of objects more accurately. Deep learning-based methods mainly use the feature extraction ability of convolutional neural networks to learn the features of various objects in the image and analyze various feature representations to obtain the edge information of objects in image features.

Researchers utilize the feature extraction capabilities of convolutional neural networks such as VGG (Simonyan and Zisserman, 2014), ResNet (He et al., 2016; Liao et al., 2025), and other backbone networks (Zhang et al., 2024; Ren et al., 2024; Wang et al., 2024; Lei et al., 2024) to design multiple deep learning architectures for object edge detection (Al-Amaren et al., 2021; Park et al., 2020). Moreover, multi-scale information in edge detection is very important. Some methods that utilize multi-scale information (Dong et al., 2022; Ma et al., 2020; Qiu et al., 2021) are applied to image processing tasks. In Dong et al. (2022), using multi-scale edge information for auxiliary training can better complete tasks such as image compression. In Ma et al. (2020), edge detection is elaborately described as edge detection, object contour detection, and semantic edge detection. Among them, edge detection mainly extracts the edges in the input image by relying on low-level, fine-grained features. Object contour detection locates the edges and suppresses those edges that do not belong to the object contour, usually requiring low-level and middle-level features. And semantic edge detection, that is, extracting object boundaries and classifying them, needs to cover features of all levels. This means that in edge detection, to achieve different results for different task goals, the influence of features at different levels must be considered.

The methods described above all start from the spatial domain information of images. However, images in the frequency domain space can also easily filter out interference and further enhance the edge information of the main body. Therefore, combining the spatial and frequency domains, this paper proposes a multi-scale edge detection network with spatial-frequency domain interactive attention (SFIA-MSNet) for accurate and stable detection of object edges in images. Two main modules are designed. The spatial-frequency domain interactive attention module can suppress the interference information in the image in the frequency domain and obtain its edge frequency information in the frequency domain, obtain the edge features of the image enhanced in the frequency domain, and interact with the original image in the spatial domain, making the object edges of the spatial domain image more significant and obtaining a higher-quality edge feature representations, so that it is easier to be detected. The multi-scale module mainly obtains the feature representations of the enhanced features at different scales, which can reflect more fine-grained edge information. After experiments on multiple datasets such as BSDS500 (Arbelaez et al., 2010), NYUDV2 (Silberman et al., 2012), and Multicue (Mély et al., 2016), and comparison with the most advanced methods, the results obtained using the proposed architecture are more accurate in quality and precision and are superior to all methods.

The chapter arrangement of this paper is as follows. Section 2 introduces related work. Section 3 presents the proposed edge detection method. Section 4 describes the settings and results of the experiment and analyzes and discusses them. Section 5 is the conclusion.

## 2 Related work

Edge detection has always been able to provide key information in many image processing tasks. Its methods are mainly divided into two categories: traditional manual edge detection and deep learning-based edge detection.

### 2.1 Traditional edge detection

Traditional edge detection algorithms are usually used for the edge detection of simple objects because they occupy less computing resources. The active contour method can detect edges in images more accurately with the help of a reasonable mathematical model. Cai et al. (2018) proposed an adaptive scale active contour model (ASACM) based on image entropy and a semi-naive Bayes classifier to achieve simultaneous segmentation field estimation. ASACM first constructs an adaptive scale operator and adjusts the scale according to the degree of non-uniformity of image intensity. Secondly, through the improved bias field estimation term, a dependent membership function is assigned to each pixel to effectively estimate the bias field. Finally, a new piecewise polynomial penalty term is introduced to avoid time-consuming reinitialization and the instability of traditional penalty terms. Ma et al. (2024) proposed an active contour model based on the Laplacian operator (LOACM). LOACM combines a global pre-fitting function constructed by using the second-order gradient information of the image with an adaptive boundary indicator function to construct a hybrid model for accurately detecting the edge information of the image.

Multi-scale information also plays a very important role in object edge detection. Different scales can further describe the edge information of an image. Cui et al. (2021) proposed a multi-scale adaptive image edge detector. Firstly, a multi-scale pyramid image is constructed. The gradient map and standard deviation map are calculated by utilizing the characteristics of the gradient and local gradient difference. Candidate edges are detected through pixel-by-pixel detection and weighted fusion. Finally, a binarized edge map is obtained through adaptive linking, which improves detection accuracy. In Li et al. (2019), proposed a method to obtain edge maps by using multi-scale anisotropic Gaussian kernels. The directional derivative is introduced to obtain multi-scale local intensity changes. Secondly, the multi-scale edge intensity map is fused to solve the contradiction between noise robustness and accurate edge extraction. Finally, the fused map is embedded in the Canny framework to obtain edge contours.

When the anisotropic diffusion model is used in image edge detection, it can obtain the attribute information of the same position from different directions, and then can more accurately describe the edge information in the image. Guo et al. (2018) proposed an improved speckle reducing anisotropic diffusion (SRAD) method. SRAD constructs a new edge detection operator by using weighted Euclidean distance. This operator can adaptively distinguish homogeneous and heterogeneous image regions, effectively generate anisotropic diffusion coefficients for each image pixel, and filter each pixel on different scales to obtain more accurate edge information. In Gupta and Lamba (2021), a new diffusion coefficient is proposed for the anisotropic diffusion model, which has a high convergence speed and image-dependent threshold parameters. This method effectively suppresses step artifacts and avoids edge blurring, and performs excellently in noise removal and edge preservation.

Researchers have also proposed a series of extensive methods by leveraging frequency domain information. In Zhang et al. (2022), combined the threshold denoising method of wavelet neural networks for edge detection. This method retains more real information and improves the impact of noise on edge images. In addition, Zheng et al. (2023) introduced an edge detection method based on a two-dimensional discrete cosine transform and frequency domain. It combines frequency domain filtering with anisotropic gradient operator and can extract the edge information of unknown objects in the case of an under-sampling rate.

### 2.2 Deep learning edge detection

With the development of computing power and deep learning, deep learning-based edge detection methods are currently more cutting-edge technologies. Through the autonomous learning ability of computers, edges in images can be quickly and accurately detected without the need to construct a mathematical model. Among them, the attention method is introduced in various image tasks, greatly improving the completion degree of each task. Liu et al. (2022) proposed an edge attention network (EdgeAtNet). When processing low-level features, EdgeAtNet inserts a global view attention block at the bottleneck of the shallow network to capture the long-range dependence of edge features. When processing high-level features, local focus attention is designed to achieve a clear boundary representation. Zhang J. et al. (2023) proposed an attention-guided edge refinement network (AERNet). AERNet uses a global context feature aggregation module to aggregate the information obtained from multi-layer context features. Combined with an attention decoding block guided by enhanced coordinate attention to capture the channel and position associations of features. It also uses an edge-refinement module to enhance the network's ability to perceive and refine the edges of changing regions.

Multi-scale information plays an important role in the learning of image edge features in deep learning networks and can further refine edge features. Therefore, multi-scale information is often introduced into the network to enhance the extraction ability of fine features. Liu et al. (2017) proposed a method using richer convolutional features (RCF). RCF encapsulates all convolutional features in more discriminative representations, making full use of the multi-scale and multi-level information of objects and comprehensively carrying out image-to-image prediction. Xuan et al. (2022) proposed a novel fine-scale correction learning network (FCL-Net). FCL-Net is mainly composed of a top-down attention-guided (TAG) module and a pixel-level weighting (PW) module. With the help of long short-term memory, the TAG module can adaptively learn and repeatedly fuse multi-scale features under the guidance of coarse-scale depth-supervised predictions, effectively promoting fine-scale feature learning. The PW module independently processes the contribution of each spatial position and helps the fine-level branch detect detailed edges with high confidence. Elharrouss et al. (2023) proposed a cascaded high-resolution network (CHRNet) to overcome the challenges of refined edge detection. This network is interconnected between successive parts of the network and uses refined batch normalization layers to maintain high-resolution edges.

Frequency domain information is different from spatial domain information and can also reflect different attributes in images. Reasonable use of frequency domain information in images can more accurately obtain the edge information of images. Guobin et al. (2020) found that noise information in images can be filtered out through wavelet transformation. A wavelet transform denoising method and morphological gradient operator are proposed. By selecting the appropriate structural elements of remote sensing images, noise pixels cannot participate in morphological calculation. The noise intensity changes with the size of the quantum superposition state structural elements, which can better filter out the noise information in the graph and enhance the image edge representations. You et al. (2023) proposed a wavelet transform algorithm that uses four wavelet functions and four decomposition levels to filter and reconstruct images. Denoising the image before edge detection can improve the signal-to-noise ratio of the image and retain as much edge information as possible.

Different from the above edge detection methods that only rely on spatial domain or frequency domain information, our research hopes to combine the features of these two domains. It can make full use of the advantages of features in different fields, use the self-attention mechanism, and take advantage of the differences between frequency domain and spatial domain features to obtain significant features, improve the accuracy after detection, and finally obtain more comprehensive and reliable results.

## 3 Methods

**Figure 1:**
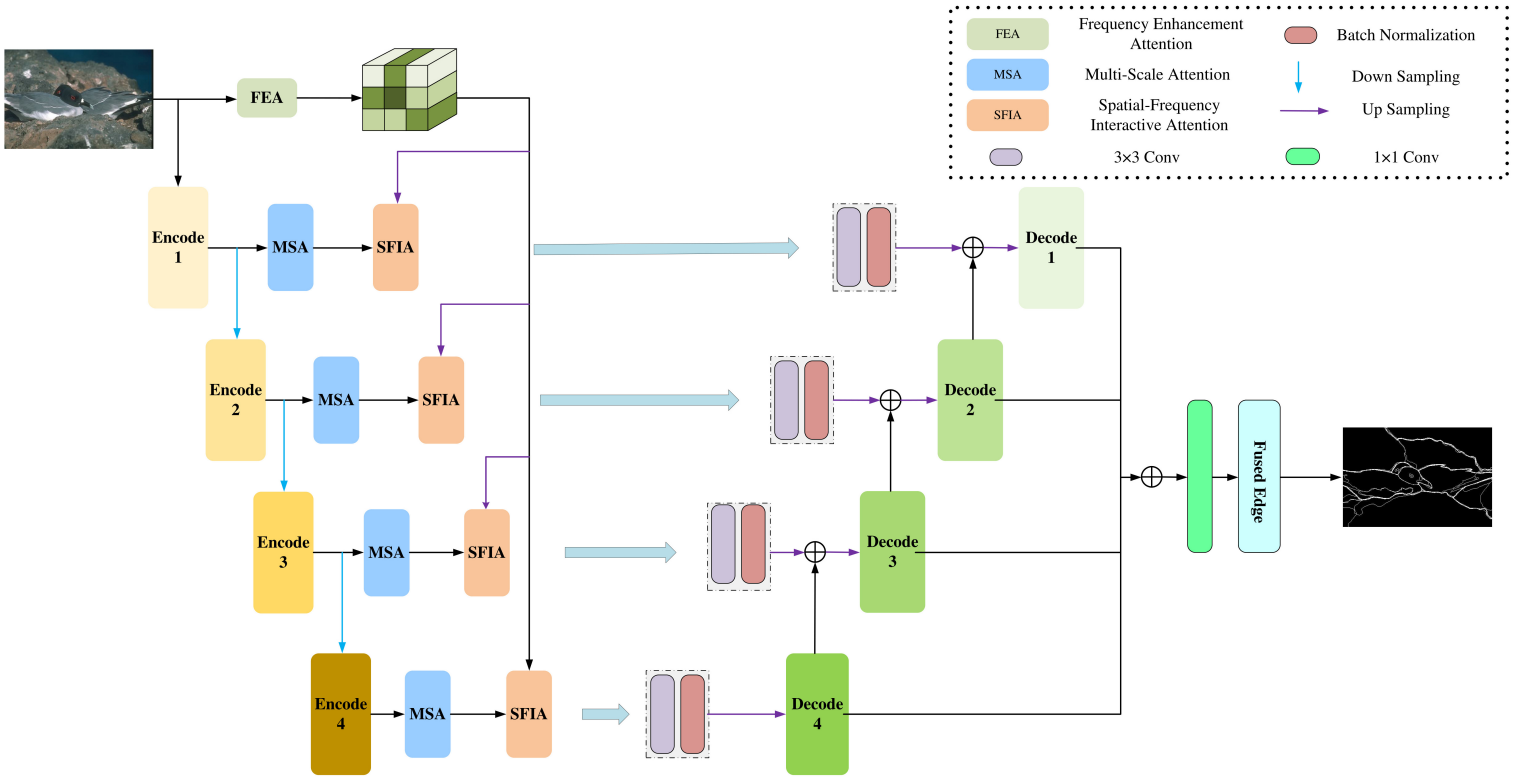
The multi-scale edge detection framework with spatial-frequency domain interactive attention proposed in this paper first extracts features from spatial domain images through Block 1 to Block 4 and uses the MSA module for spatial domain enhancement. Then, it fuses the frequency domain features after frequency domain enhancement with the spatial domain features obtained from each Block to obtain more accurate features. Finally, the edge map is output through the decoder.

To obtain clearer and more accurate edges, we propose a multi-scale edge detection network method with spatial-frequency domain interactive attention. Figure 1 is the framework of our method, including three modules: using the frequency enhancement attention (FEA) module for extracting significant frequency domain edge features; using the multi-scale attention (MSA) module for extracting multi-scale features in the spatial domain; using the spatial-frequency interactive attention (SFIA) module for interactive attention between frequency domain features and spatial domain features to obtain the spatial domain features enhanced by the frequency domain. Next, we will introduce them in detail.

### 3.1 Frequency enhancement attention module

The information of the image in the spatial domain is clearly visible. However, due to the limitation that certain specific features cannot be separated in the spatial domain, frequency domain features are introduced in this research to improve the ability to obtain edge information in complex images. Therefore, in order to better utilize frequency characteristics, this paper proposes a novel FEA module to obtain significant edge information in the frequency domain space. It is mainly divided into two parts. First, obtain the low-frequency and high-frequency information of the image in the frequency domain space. Secondly, enhance the frequency domain information of the edge, and use the self-attention mechanism to obtain the correlation between high and low frequencies to further filter out the edge information of the background.

**Figure 2:**
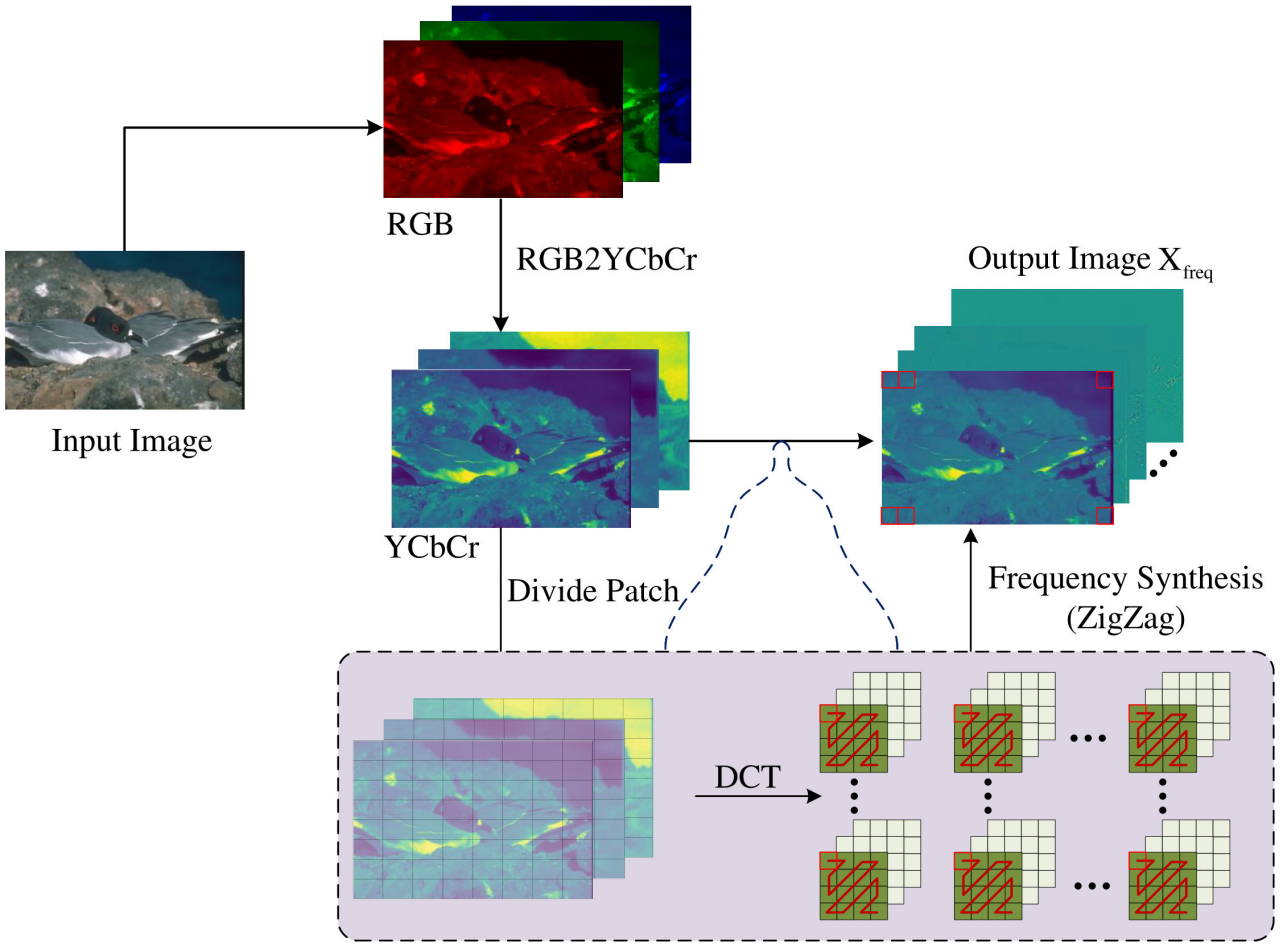
The low-frequency and high-frequency information in the frequency domain is separated by the ZigZag method after the input RGB image is cut into patches and undergoes DCT transformation.

The acquisition of frequency domain information is obtained by performing specific transformations on the image in the spatial domain to obtain the frequency domain information of the image. Under normal circumstances, doing so can divide the image information in the frequency domain space into low-frequency information and high-frequency information. Compared with the spatial domain, the frequency domain can divide the features that are difficult to separate in the spatial domain to a certain extent so that operations can be performed for the required frequency band to complete certain specific image tasks. As shown in Figure 2, in this paper, discrete cosine transform (DCT) (Ahmed et al., 1974) is used to obtain image frequency domain information because DCT transform can effectively compress image information and at the same time remove the correlation between image pixels, making edges easier to be detected. After the image is transformed by DCT, ZigZag (Al-Ani and Awad, 2013) is used to divide the frequency signal into high-frequency and low-frequency components. First, convert the RGB image X of 3 × *H* × *W* pixels into the YCbCr domain, and then divide it into $\frac{H}{8}\times \frac{W}{8}$ image blocks $X_{patch}^{i}$ of 8 × 8 pixels in each channel, and perform DCT transformation to obtain $X_{t}^{i}$(*i* = 1, 2, 3), i refers to the number of channels. In the ZigZag method, a 1 × 1 window is used to slide in a zigzag direction in each image block to sort the frequencies of each $X_{t}^{i}$(*i* = 1, 2, 3). The similar frequency signals of $X_{t}^{i}$(*i* = 1, 2, 3) in the Y, Cb, and Cr channels are recombined into a new feature map. The grouped feature maps are sorted from low to high in frequency, which can effectively separate the high-frequency and low-frequency components from the original feature map so that each new feature map only contains similar low-frequency and high-frequency bands. The first 32 dimensions obtained under each channel are low-frequency components $X_{low}^{i}$, and the last 32 dimensions are high-frequency components $X_{high}^{i}$. The low-frequency and high-frequency information obtained from the three channels are spliced respectively to obtain the low-frequency information *X*_*low*_ and high-frequency information *X*_*high*_ of the image:


(1) $$\begin{array}{l} \begin{array}{l} X_{t}^{i}=DCT\left(X_{patch}^{i}\right),\\ X_{low}^{i},X_{high}^{i}=ZigZag\left(X_{t}^{i}\right),\\ X_{low}=Concat\left(X_{low}^{i}\right),i\in \left[0,31\right],\\ X_{high}=Concat\left(X_{high}^{i}\right),i\in \left[32,63\right]. \end{array} \end{array}$$

**Figure 3:**
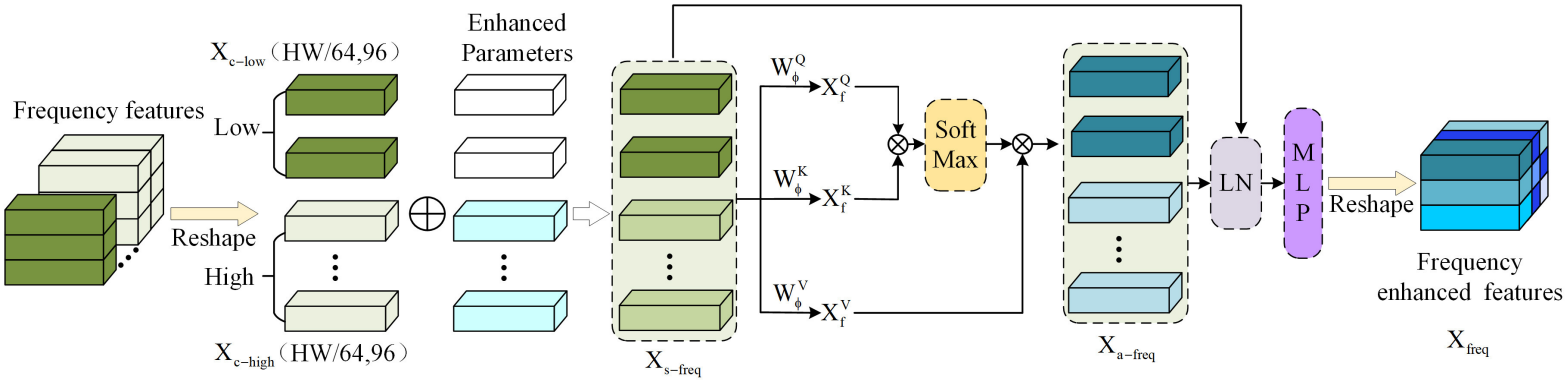
Obtain significant edge information by using self-attention in frequency domain information.

After obtaining the separated low-frequency and high-frequency image information using the DCT and ZigZag methods. As shown in Figure 3, the self-attention mechanism is used to interact with the low-frequency and high-frequency information to obtain the correlation between frequency domains and further enhance the frequency domain information of the image edge. Since the self-attention focuses on the correlation between image blocks when in use, before splicing, the low-frequency information *X*_*low*_ and the high-frequency information *X*_*high*_ are reshaped from the shape of $\left(1,96,\frac{H}{8},\frac{W}{8}\right)$ to $\left(1,\frac{HW}{64},96\right)$, denoted as *X*_*c*−*low*_ and *X*_*c*−*high*_. Since the edge information is generally preserved in the high frequency, an enhancement parameter ρ is set to fuse it into the high-frequency information to obtain the enhanced high-frequency edge information *X*_*s*−*high*_. Secondly, the low-frequency information *X*_*c*−*low*_ and the high-frequency information *X*_*s*−*high*_ are spliced into a complete frequency domain information *X*_*s*−*freq*_ using Concat. The output of FEA is calculated based on the standard self-attention operation in the transformer encoder. The calculation operation is as follows:


(2) $$\begin{array}{l} Attention\left(Q,K,V\right)=SoftMax\left(\frac{QK^{T}}{\sqrt{d^{k}}}\right)V \end{array}$$

Therefore, the *X*_*a*−*freq*_ after being enhanced by self-attention is:


(3) $$\begin{array}{l} X_{a-freq}=Attention\left(X_{s-freq}W_{\phi }^{Q},X_{s-freq}W_{\phi }^{K},X_{s-freq}W_{\phi }^{V}\right),\\ \;X_{a-freq}\in R^{\left(\frac{HW}{64}\times 192\right)}. \end{array}$$

Among them, $W_{\phi }^{Q}$, $W_{\phi }^{K}$, and $W_{\phi }^{V}$ are a set of learnable weight parameters. Next, continuous calculations are performed through layer normalization (LN) and multilayer perceptron (MLP):


(4) $$\begin{array}{l} X_{freq}=MLP\left(LN\left(X_{a-freq}+X_{s-freq}\right)\right). \end{array}$$

### 3.2 Multi-scale attention module

In image edge detection, there may be a problem that the main target is too small, resulting in inaccurate detection. In response to this, this paper proposes the MSA module, which enables the network to accurately locate and detect relatively fine edge features by expanding the receptive field.

**Figure 4:**
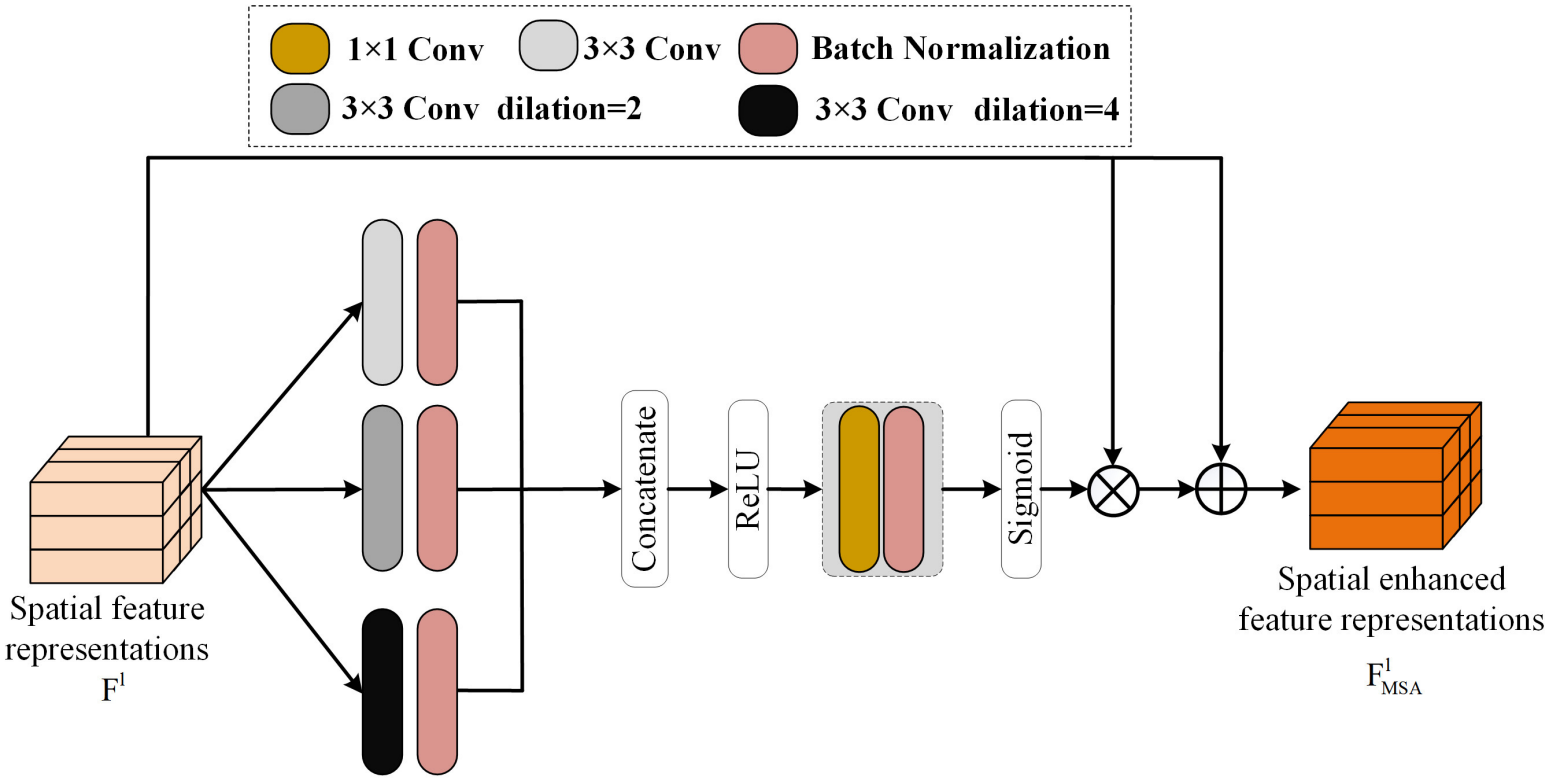
The multi-scale attention module proposed in this paper can obtain fine features under different receptive fields by making the input features pass through dilated convolutions with different dilation rates respectively. And by using the splicing and SoftMax operations to obtain the attention weights of each channel, it can obtain the fine edge features of the input features at different scales.

In this research, ResNet50 (He et al., 2016) is adopted as the backbone network in the spatial domain and used as the encoder for image feature extraction. The ResNet50 network has four Blocks, and each Block serves as an encoder to extract features of the image at the current scale. After the input image *X* passes through the four encoders composed of the ResNet50 network, the feature representation *F*^*l*^ (*l* = 1, 2, 3, 4) at the current scale is obtained. After the feature output *F*^*l*^ passes through the MSA module, more refined feature representations at the current scale can be obtained. In the MSA module, dilation convolution is introduced with magnification factors of 2 and 4. As shown in the Figure 4, after the feature representations *F*^*l*^ pass through convolutions with magnification factors of 1, 2, and 4, respectively, it is integrated into a feature representation with three times the original number of channels through splicing. Then, through convolution with a kernel size of 1, its number of channels is changed to the original number of channels. Softmax is used to obtain the weight parameters of the channels and multiply them with the original features. This can be regarded as a kind of channel attention. Finally, it is fused with the original features through the residual method, which can obtain fine features while avoiding discarding useful feature information. The processing of the MSA module can be expressed as:


(5) $$\begin{array}{l} \begin{array}{l} F_{c}^{l}=Concat\left(F_{1}^{l},F_{2}^{l},F_{4}^{l}\right),\\ F_{\omega }^{l}=SoftMax\left(F_{c}^{l}\right),\\ F_{MSA}^{l}=F^{l}+\left(F^{l}\times F_{\omega }^{l}\right). \end{array} \end{array}$$

Among them, $F_{1}^{l}$, $F_{2}^{l}$, and $F_{4}^{l}$ respectively represent the results obtained by *F*^*l*^ passing through dilation convolutions with different magnification factors and a convolution kernel size of 3 × 3. $F_{MSA}^{l}$ represents the final output of the feature representations of the *l* − *th* block in the ResNet12 network after passing through the MSA module. The MSA module plays a huge role in increasing the receptive field of the network. After using the MSA module, the network captures more edge information, thus retaining a large amount of edge information.

### 3.3 Spatial-frequency interactive attention module

**Figure 5:**
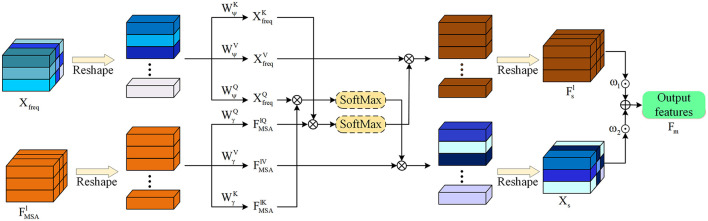
Use spatial-frequency domain interactive attention to mutually enhance and fuse frequency domain features and spatial domain features to obtain more accurate edge information.

After obtaining the feature representations of the image in both the spatial domain and the frequency domain, further consideration needs to be given to how to use the spatial and frequency domain feature representations to further strengthen the edge information of the image. Generally speaking, simple addition after feature alignment can make the frequency domain and spatial domain features fuse with each other, but doing so cannot find information more conducive to image edge detection from the spatial domain or frequency domain. In order to be able to use the features of the spatial and frequency domains to more accurately detect the edge features of the image. As shown in the Figure 5, this paper designs the SFIA module. Through mutual attention, the frequency domain and spatial domain features of the image interact. The spatial domain features are used to guide and enhance the frequency domain, and the frequency domain features are used to guide and enhance the spatial domain features, respectively. Finally, the enhanced spatial domain features and frequency domain features are fused to realize the interaction between the spatial domain features and frequency domain features of the image and enhance the edge information. Before the frequency domain features are sent to the SFIA module, since the size of the current frequency domain feature map is $\frac{H}{8}\times \frac{W}{8}$, which is the same as the size of the output of the last Encode of the spatial domain features, when performing SFIA at each layer, first perform sampling and other operations on the frequency domain feature map to obtain the transformed frequency domain feature *X*_*freq*_, so that the shape of the frequency domain feature is consistent with that of the spatial domain.

Specifically, the SFIA module introduces interactive attention. The enhanced frequency domain feature *X*_*freq*_ is multiplied by the learnable weight parameters $W_{\psi }^{Q}$, $W_{\psi }^{K}$, and $W_{\psi }^{V}$ to obtain *X*_*q*−*freq*_, *X*_*k*−*freq*_, and *X*_*v*−*freq*_ respectively. The enhanced spatial domain feature $F_{MSA}^{l}$ of each layer is multiplied by the learnable weight parameters $W_{\gamma }^{Q}$, $W_{\gamma }^{K}$, and $W_{\gamma }^{V}$ to obtain $F_{q-MSA}^{l}$, $F_{k-MSA}^{l}$, and $F_{v-MSA}^{l}$ respectively. The frequency domain feature *X*_*s*_ enhanced by the spatial domain and the spatial domain feature $F_{s}^{l}$ enhanced by the frequency domain are calculated respectively through the formulas described below. The final output feature of each layer $F_{m}^{l}$ is obtained by weighted summation of the frequency domain feature *X*_*s*_ and the spatial domain feature $F_{s}^{l}$, and is represented as:


(6) $$\begin{array}{l} \begin{array}{l} X_{s}=Attention\left(X_{freq}W_{\psi }^{Q},X_{freq}W_{\psi }^{K},X_{freq}W_{\psi }^{V}\right),\\ F_{s}^{l}=Attention\left(F_{MSA}^{l}W_{\gamma }^{Q},F_{MSA}^{l}W_{\gamma }^{K},F_{MSA}^{l}W_{\gamma }^{V}\right),\\ F_{m}^{l}=\omega_{1}\times X_{s}+\omega_{2}\times F_{s}^{l}. \end{array} \end{array}$$

Here, Attention(·) is as shown in Equation 2.

### 3.4 Loss function

For a typical edge image, the ratio of non-edge pixels to edge pixels is extremely imbalanced. Around 90% of the pixels in the true label map are non-edge pixels. Hence, we use a weighted cross-entropy loss for each edge map. Given a predicted edge map *F* = {*f*_*i*_ ∈ [0, 1], *i* = 1, 2, ⋯ , |*F*|} and its corresponding ground truth *Y* = {*y*_*i*_ = 0 *or* 1, *i* = 1, 2, ⋯ , |*Y*|}, the calculation formula for the loss function *L* is as follows:


(7) $$\begin{array}{l} L=loss\left(F,Y\right)=-\alpha \underset{y_{i}\in Y_{+}}{\sum }log\left(f_{i}\right)-\beta \underset{y_{i}\in Y_{-}}{\sum }log\left(1-f_{i}\right). \end{array}$$

where $\alpha =\frac{\left|Y_{-}\right|}{\left|Y_{+}|+|Y_{-}\right|}$, $\beta =\lambda \frac{\left|Y_{+}\right|}{\left|Y_{+}|+|Y_{-}\right|}$. Usually, edge detection datasets will have multiple different annotations. However, due to subjective ambiguity, these annotations often have deviations in aspects such as location, even edges, or non-edge pixels. This inconsistency will bring a lot of noise and mislead optimization during the training process. In order to reduce this inconsistency or error, a threshold θ is set to filter out annotations that may be incorrect. The positive edge is defined as *Y*_+_ = {*y*|*y* ≥ θ}, and *Y*_−_ = {*y*|*y* = 0} represents the negative edge. The coefficient λ is a learnable parameter whose role is to adjust the weights of edge and non-edge pixels.

## 4 Experiments and result analysis

### 4.1 Datasets

In this section, we will evaluate the performance of our proposed method on three benchmark datasets, including BSDS500 (Arbelaez et al., 2010), Multicue (Mély et al., 2016), and NYUDV2 (Silberman et al., 2012), and compare it with previous advanced methods.

BSDS500 is a benchmark dataset created by the Berkeley Center for Computer Vision and Machine Learning for image segmentation and edge detection. It contains 500 natural images. In the experiment, 200 images are used as the training set, 100 images as the validation set, and the remaining 200 images as the test set.

Multicue contains 100 challenging natural images, each of which contains a binocular view. Boundaries and edges are strictly distinguished in the images. Among them, boundaries are the boundary pixels of meaningful objects, while edges refer to sudden changes related to brightness, color, and texture. They are randomly divided into 80 images for training and 20 images for testing, and the performance of the boundary subset and the edge subset is evaluated separately.

NYUDV2 is a commonly used dataset for deep learning and computer vision research, mainly used for semantic segmentation and depth estimation tasks of indoor scenes. There are a total of 1449 RGBD images of 640 × 480 pixels.

### 4.2 Implementation details

In the experiment, we use the deep learning framework PyTorch to train the method proposed in this paper. ResNet50 is selected as the network backbone. The Adam algorithm is used as the optimizer, and the batch size is set to 8. During the training process, the epoch is set to 200, and the global learning rate is 1 × 10^−5^. All experiments are completed on an NVIDIA RTX3090Ti GPU. In the experiment, operations such as rotation, scaling, flipping, and cropping are applied for data augmentation. After augmentation, the number of images in the BSDS500 training set is expanded to 28,800. Similarly, through data augmentation, the training sets of the Multicue and NYUDV2 datasets reach 11,520 and 54,864 images, respectively. In the experiment, the Optimal Image Scale (OIS), Optimal Dataset Scale (ODS), and Average Precision (AP) were used as performance indicators to evaluate the advantages and disadvantages of the method proposed in this paper and other methods.

### 4.3 Ablation study

In this subsection, quantitative analysis is conducted on the FEA, MSA, and SFIA modules proposed in this paper on the BSDS500 dataset respectively to verify the effectiveness of each module proposed in this article for edge detection. In the evaluation stage, we use non-maximum suppression (NMS) technology to thin and normalize the detected edges.

**Figure 6:**
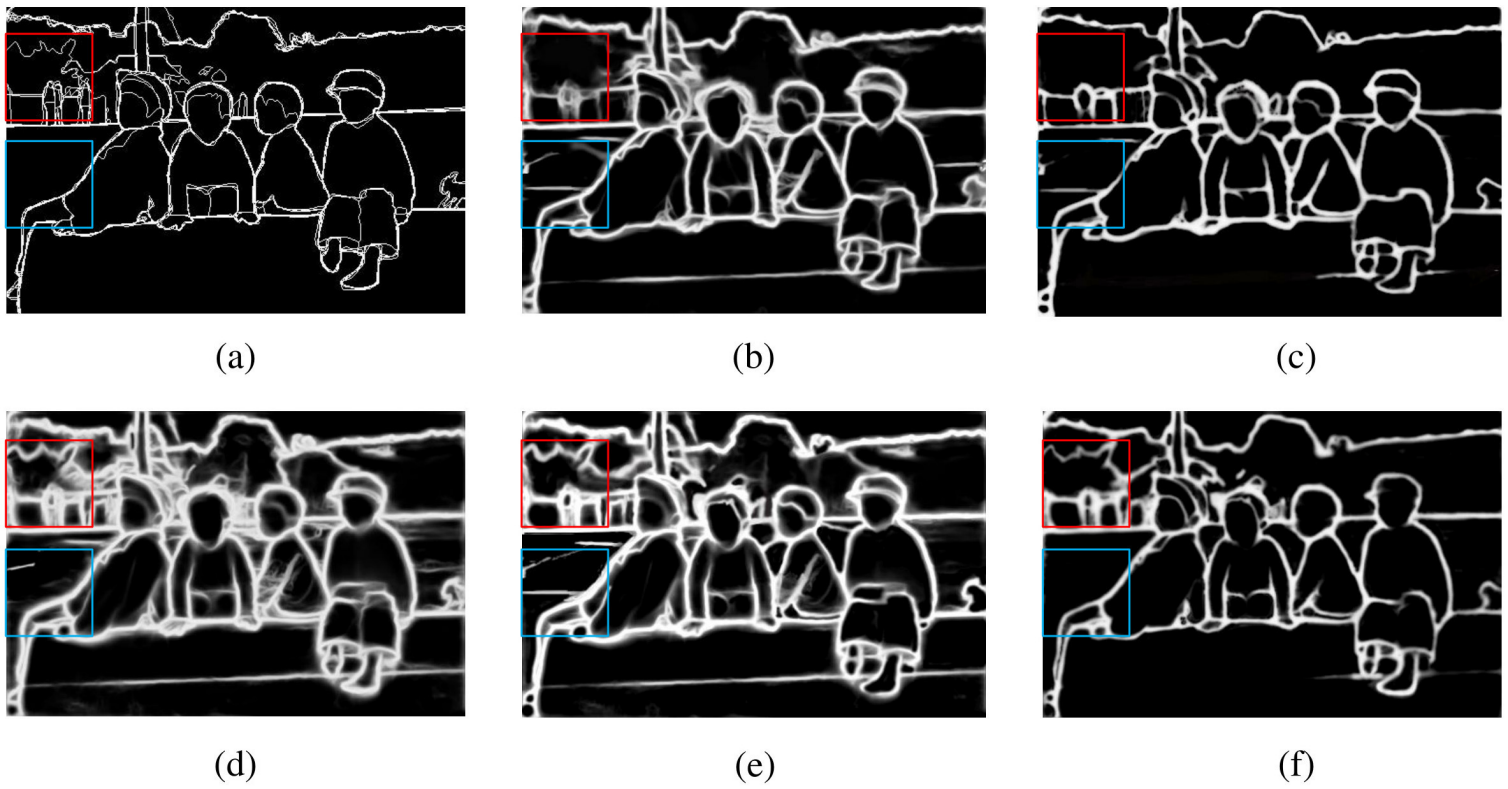
Ablation study under the BSDS500 dataset, **(a)** Ground truth map, **(b)** Baseline network, **(c)** Baseline network + MSA module, **(d)** Baseline network + FEA module, **(e)** Baseline network + MSA + FEA module, **(f)** Baseline network + MSA + FEA + SFIA module.

For the ground truth maps of some images in the dataset, as shown in Figure 6a, they are compared with the edge maps obtained by other methods for analysis. As can be seen from Figure 6b, when only the baseline network is used, not only will a large amount of edge information be lost, but the edges are also relatively rough. In Figure 6c, after introducing the MSA module into the baseline network, some fine features can also be detected, and the detected edges are also more refined. In Figure 6d, when the FEA module is introduced into the baseline network, it can be found that the edges in the background other than the larger main body can also be detected, but at the same time, some interfering edge information is also detected, and the edges are rough. In Figure 6e, when the MSA and FEA modules are directly combined and used, the detection refinement is enhanced, but there are still some errors. When the SFIA module is used to mutually enhance the two features, it can be found in Figure 6f that not only can relatively fine edges be obtained, but also the interference information is greatly reduced. It can also be clearly observed from Table 1 that when the three modules of MSA, FEA, and SFIA proposed in this paper are introduced into the baseline network, there are improvements in all three indicators, which also shows the effectiveness of the SFIA-MSNet proposed in this paper.

**Table 1:** The results of validating the effectiveness of multiple modules proposed in this paper on the BSDS500 dataset respectively.

**Baseline**	**FEA**	**MSA**	**SFIA**	**ODS**	**OIS**	**AP**
✓	×	×	×	0.773	0.791	0.813
✓	✓	×	×	0.798	0.815	0.856
✓	×	✓	×	0.812	0.833	0.858
✓	✓	✓	×	0.817	0.841	0.869
✓	✓	✓	✓	0.824	0.845	0.885

### 4.4 Comparison with the state-of-the-arts

In this section, the performance of the method proposed in this paper is compared with that of multiple advanced methods under the official standard dataset. At the same time, the differences in the results of some model visualizations are analyzed.

#### 4.4.1 Performance on BSDS500 dataset

**Figure 7:**
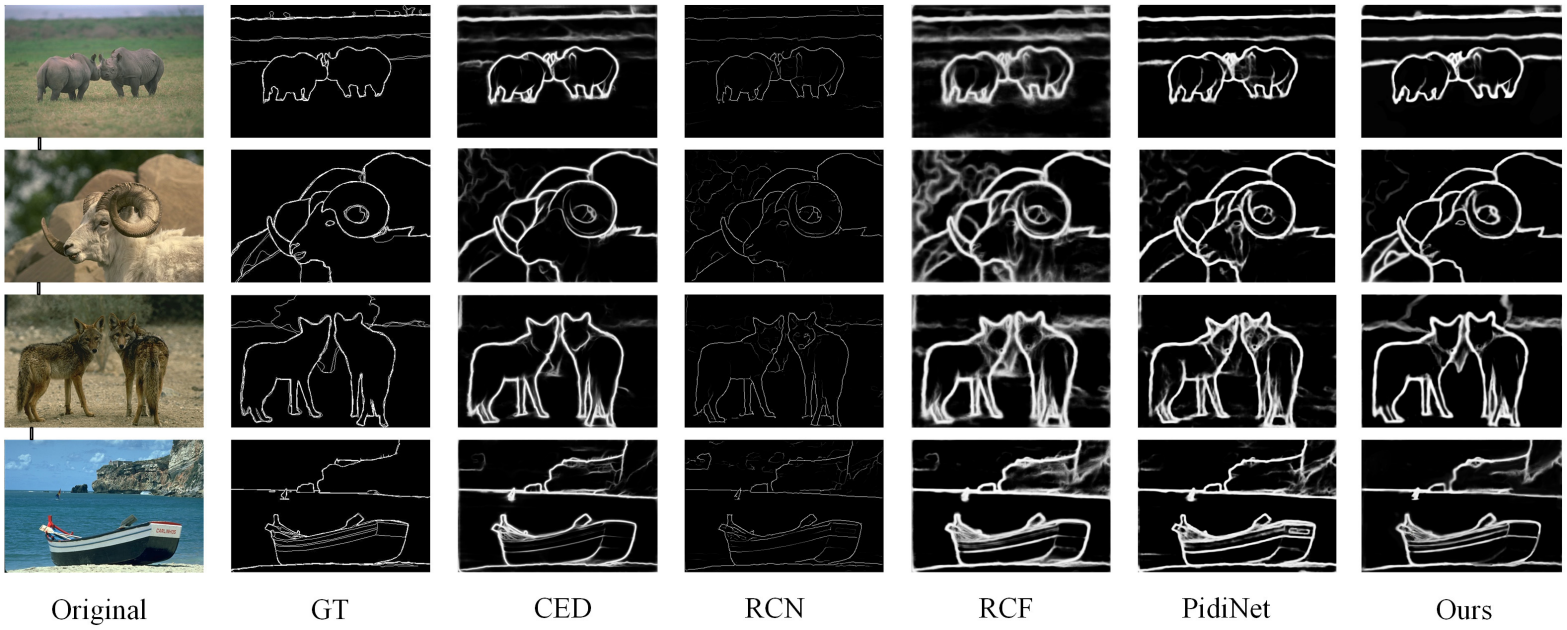
Some visualization results of edge detection on the BSDS500 dataset.

Performance on BSDS500 dataset: The method SFIA-MSNet proposed in this paper is compared with several relatively excellent methods, including DeepEdge (Bertasius et al., 2015), CED (Wang et al., 2017), HED (Xie and Tu, 2017), LPCB (Deng et al., 2018), RCN (Kelm et al., 2019), RCF (Liu et al., 2019), REDN (Le and Duan, 2020), PiDiNet (Su et al., 2021), RHN (Al-Amaren et al., 2021), BDCN (He et al., 2022), EDTER (Pu et al., 2022), DexiNed (Soria et al., 2023), CHRNet (Elharrouss et al., 2023), DiffusionEdge (Ye et al., 2024) and FF-CNSNP (Xian et al., 2024). In Figure 7 also shows the visualization results of some algorithms and the SFIA-MSNet method in the BSDS500 dataset. As can be seen from Table 2, under the three performance indicators of ODS, OIS, and AP, the method proposed in this paper achieves the best results.

**Table 2:** Comparison with other edge detection methods on the BSDS500 dataset, where methods denoted with “-VOC” were trained on a mixed dataset combining BSDS500 and Pascal VOC.

**Method**	**Year**	**Backbone**	**OIS**	**ODS**	**AP**
DeepEdge (Bertasius et al., 2015)	2015	AlexNet	0.772	0.753	0.807
CED (Wang et al., 2017)	2017	VGG16	0.811	0.794	0.847
HED (Xie and Tu, 2017)	2017	VGG16	0.808	0.788	0.840
LPCB (Deng et al., 2018)	2018	VGG16	0.816	0.800	-
RCN (Kelm et al., 2019)	2019	ResNet	0.838	0.823	0.853
RCF (Liu et al., 2019)	2019	VGG16	0.815	0.798	-
REDN (Le and Duan, 2020)	2020	DenseNet	0.828	0.808	0.827
PiDiNet-VOC (Su et al., 2021)	2021	-	0.823	0.807	-
RHN (Al-Amaren et al., 2021)	2021	VGG16	0.833	0.817	-
BDCN (He et al., 2022)	2022	VGG16	0.826	0.806	0.847
EDTER (Pu et al., 2022)	2022	ViT	0.841	0.824	0.880
DexiNed (Soria et al., 2023)	2023	-	0.745	0.729	0.583
CHRNet-VOC (Elharrouss et al., 2023)	2023	-	0.788	0.787	0.801
DiffusionEdge (Ye et al., 2024)	2024	ResNet101	0.848	0.834	-
FF-CNSNP (Xian et al., 2024)	2024	VGG16	0.827	0.808	-
SFIA-MSNet (ours)	2024	ResNet50	0.845	0.824	0.885

The method proposed in this paper reaches 0.824 on ODS, 0.845 on OIS, and 0.885 on AP. It ranks second - highest among all methods on these three metrics In the Figure 7, it can be observed that compared with the edge maps obtained by methods such as CED (Wang et al., 2017), RCF (Liu et al., 2019), RCN (Kelm et al., 2019) and PidiNet (Su et al., 2021), the method proposed in this paper is more refined, accurate on the edges, and has less background interference.

#### 4.4.2 Performance on NYUDV2 dataset

Performance on NYUDV2 dataset: The method SFIA-MSNet proposed in this paper is compared with several relatively excellent methods in the NYUDV2 dataset, including HED (Xie and Tu, 2017), LPCB (Deng et al., 2018), RCF (Liu et al., 2019), PiDiNet (Su et al., 2021), RHN (Al-Amaren et al., 2021), BDCN (He et al., 2022), EDTER (Pu et al., 2022), CHRNet (Elharrouss et al., 2023), DiffusionEdge (Ye et al., 2024) and FF-CNSNP (Xian et al., 2024). The results of the SFIA-MSNet method and other detection models are shown in Table 3.

**Table 3:** Comparison with other edge detection methods on the NYUDV2 dataset.

**Method**	**Year**	**Backbone**	**OIS**	**ODS**	**AP**
HED (Xie and Tu, 2017)	2017	VGG16	0.734	0.720	0.734
LPCB (Deng et al., 2018)	2018	VGG16	0.754	0.739	-
RCF (Liu et al., 2019)	2019	VGG16	0.757	0.743	-
PiDiNet (Su et al., 2021)	2021	-	0.747	0.733	0.765
RHN (Al-Amaren et al., 2021)	2021	VGG16	0.762	0.751	-
BDCN (He et al., 2022)	2022	VGG16	0.763	0.748	0.770
EDTER (Pu et al., 2022)	2022	ViT	0.789	0.774	0.797
CHRNet (Elharrouss et al., 2023)	2023	-	0.745	0.729	0.818
DiffusionEdge (Ye et al., 2024)	2024	ResNet101	0.766	0.761	-
FF-CNSNP (Xian et al., 2024)	2024	VGG16	0.754	0.741	-
SFIA-MSNet (ours)	2024	ResNet50	0.791	0.775	0.798

**Figure 8:**
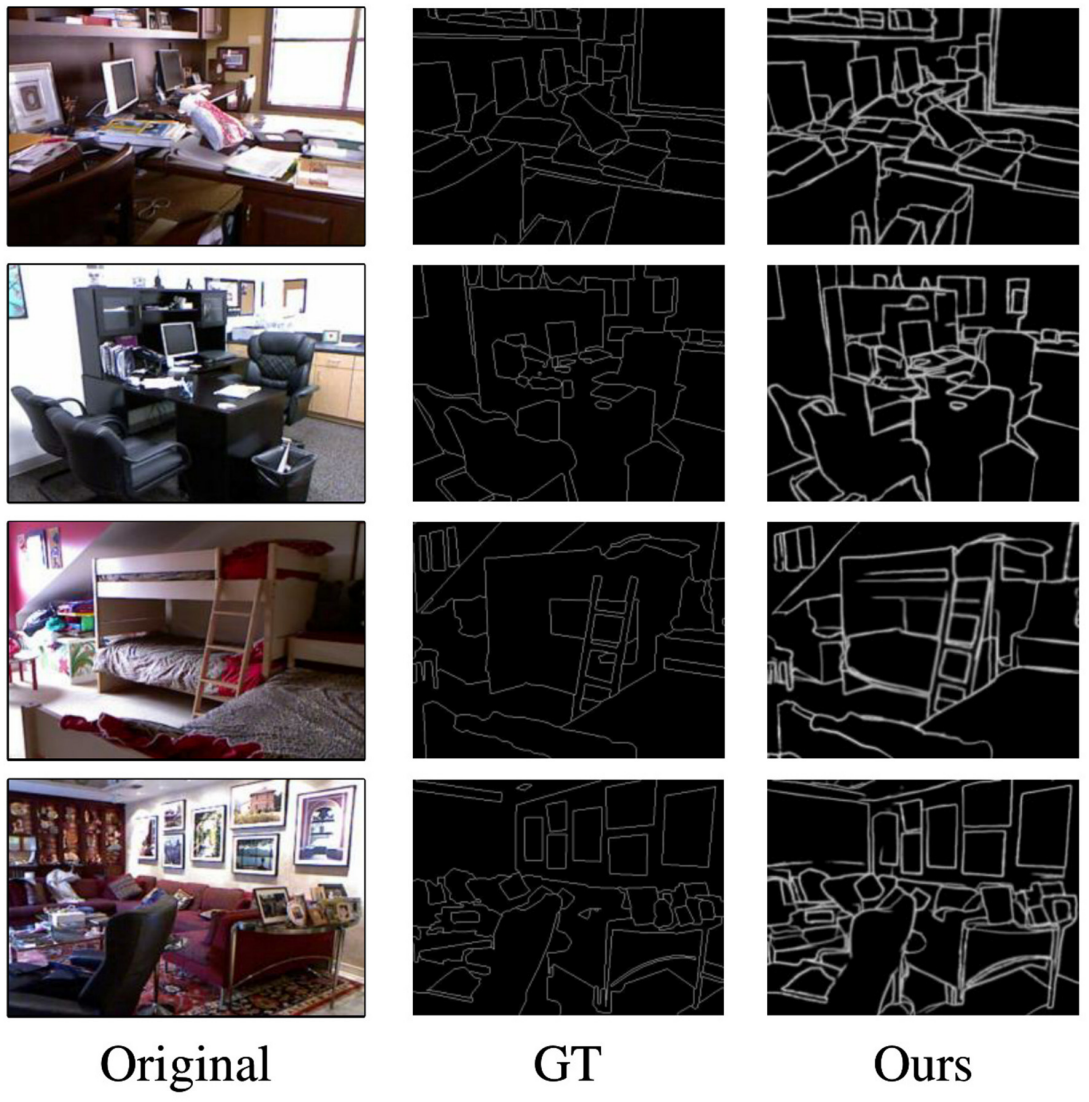
Some visualization results of edge detection on the NYUDV2 dataset.

As can be seen from the Table 3, the performance of SFIA-MSNet is still the best, with ODS of 0.775, OIS of 0.791, and AP of 0.798. Compared with the method DiffusionEdge (Ye et al., 2024) and FF-CNSNP (Xian et al., 2024), the ODS, OIS, and AP of SFIA-MSNet are all improved. Figure 8 shows the visualization results of other models such as CED (Wang et al., 2017), RCF (Liu et al., 2019), RCN (Kelm et al., 2019) and PidiNet (Su et al., 2021), and the SFIA-MSNet method on NYUDV2. From these results, it can be seen that even in complex indoor scenes, the SFIA-MSNet method can also detect the subtle edges existing in the image, including the edges in the shadows, which also shows that the method proposed in this paper can detect various complex edges with high quality in the edge detection task.

#### 4.4.3 Performance on multicue dataset

Performance on Multicue dataset:The SFIA-MSNet method proposed in this paper is compared with several relatively excellent methods under two annotations (boundary and edge) in the Multicue dataset, including HED (Xie and Tu, 2017), RCF (Liu et al., 2019), PiDiNet (Su et al., 2021), RHN (Al-Amaren et al., 2021), BDCN (He et al., 2022), and EDTER (Pu et al., 2022). The results of the SFIA-MSNet method and other detection models are shown in Table 4.

**Table 4:** Comparison with other edge detection methods on the multicue dataset.

**Method**	**Year**	**Backbone**	**Boundary**			**Edge**		
			**OIS**	**ODS**	**AP**	**OIS**	**ODS**	**AP**
HED (Xie and Tu, 2017)	2017	VGG16	0.822	0.814	0.869	0.864	0.851	-
RCF (Liu et al., 2019)	2019	VGG16	0.825	0.817	-	0.862	0.857	-
PiDiNet (Su et al., 2021)	2021	-	0.830	0.818	-	0.860	0.855	-
RHN (Al-Amaren et al., 2021)	2021	VGG16	0.856	0.841	-	0.905	0.896	-
BDCN (He et al., 2022)	2022	VGG16	0.846	0.836	0.893	0.898	0.981	0.935
EDTER (Pu et al., 2022)	2022	ViT	0.870	0.861	0.919	0.900	0.894	0.944
SFIA-MSNet (ours)	2024	ResNet50	0.873	0.862	0.924	0.901	0.900	0.950

As can be seen from Table 4, the method SFIA-MSNet proposed in this paper achieves 0.873, 0.862, 0.924 and 0.901, 0.900, 0.950 for OIS, ODS, and AP respectively under the two annotations of boundary and edge. Compared with the relatively advanced method EDTER, it is superior to this method in multiple performance indicators, which also shows the superiority of the performance of the method proposed in this paper.

#### 4.4.4 Complexity analysis

To compare the complexity of SFIA-MSNet when using different backbones and methods, we compared the number of parameters and the number of FLOPs of SFIA-MSNet using different backbones and methods, as shown in Table 5.

**Table 5:** Comparison of the complexity between SFIA-MSNet with different backbones and the BDCN method.

**Method**	**Backbone**	**Parameters(M)**	**FLOPs(M)**
BDCN (He et al., 2022)	VGG16	14.71	56,658.49
SFIA-MSNet (ours)	ResNet50	32.59	20,608.97
SFIA-MSNet (ours)	VGG16	20.94	89,000.67

As shown in Table 5: Different backbones significantly impact model complexity. Take SFIA-MSNet as an example–when using the ResNet50 backbone, the number of parameters is 32.59*M* and the FLOPs reach 20, 608.97*M*. After changing to the VGG16 backbone, the parameters decrease to 20.94*M* and the FLOPs are greatly reduced to 89, 000.67*M*, indicating that the choice of backbone directly affects the model's parameter scale and computational volume. SFIA-MSNet with the VGG16 backbone has more advantages in complexity control. Cross-method comparison also highlights differences: although BDCN with the VGG16 backbone has a parameter number of 14.71*M* (lower than SFIA-MSNet's 20.94*M* with the VGG16 backbone), its FLOPs reach 56, 658.49*M*–far higher than SFIA-MSNet's 89, 000.67*M* with the VGG16 backbone. This demonstrates that when different methods use the same backbone, network structure design significantly influences computational complexity, while SFIA-MSNet (with the VGG16 backbone) shows unique optimization effects in balancing parameters and computational volume.

## 5 Conclusion

In this study, we propose an SFIA-MSNet method that combines the spatial domain and the frequency domain. It not only filters interference information in the frequency domain to obtain significant edge information but also obtains multi-scale attention features by designing the MSA module in the spatial domain. Finally, the SFIA module is used to enable the interaction between spatial domain information and frequency domain information and obtain enhanced fused information, so as to solve the problem that existing edge detection methods cannot accurately detect the edges of small objects hidden in the background. At the same time, it also effectively suppresses noise interference. The results under multiple datasets indicate that the method proposed in this paper can accurately identify and detect fine edges.

## Data Availability

The raw data supporting the conclusions of this article will be made available by the authors, without undue reservation.

## References

[B1] Ahmed N. Natarajan T. Rao K. R. (1974). Discrete cosine transform. IEEE Trans. Comp. 100, 90–93. 10.1109/T-C.1974.223784

[B2] Al-Amaren A. Ahmad M. O. Swamy M. (2021). Rhn: A residual holistic neural network for edge detection. IEEE Access 9, 74646–74658. 10.1109/ACCESS.2021.3078411

[B3] Al-Ani M. S. Awad F. H. (2013). The jpeg image compression algorithm. Int. J. Adv. Eng. Technol. 6, 1055–1062.

[B4] Amit Y. Felzenszwalb P. Girshick R. (2021). “Object detection,” in Computer Vision: A Reference Guide (Cham: Springer), 875–883.

[B5] Arbelaez P. Maire M. Fowlkes C. Malik J. (2010). Contour detection and hierarchical image segmentation. IEEE Trans. Pattern Anal. Mach. Intell. 33, 898–916. 10.1109/TPAMI.2010.161 20733228

[B6] Bertasius G. Shi J. Torresani L. (2015). “DeepEdge: a multi-scale bifurcated deep network for top-down contour detection,” in Proceedings of the IEEE Conference on Computer Vision and Pattern Recognition, 4380–4389. 10.1109/CVPR.2015.7299067

[B7] Cai Q. Liu H. Zhou S. Sun J. Li J. (2018). An adaptive-scale active contour model for inhomogeneous image segmentation and bias field estimation. Pattern Recognit. 82, 79–93. 10.1016/j.patcog.2018.05.008

[B8] Cui Y. An Y. Sun W. Hu H. Song X. (2021). Multiscale adaptive edge detector for images based on a novel standard deviation map. IEEE Trans. Instrum. Meas. 70, 1–13. 10.1109/TIM.2021.308388833776080

[B9] Deng R. Shen C. Liu S. Wang H. Liu X. (2018). “Learning to predict crisp boundaries,” in Proceedings of the European Conference on Computer Vision (Cham: Springer), 562–578.

[B10] Dong C. Chen X. Hu R. Cao J. Li X. (2022). Mvss-net: Multi-view multi-scale supervised networks for image manipulation detection. IEEE Trans. Pattern Anal. Mach. Intell. 45, 3539–3553. 10.1109/TPAMI.2022.3180556 35671312

[B11] Elharrouss O. Hmamouche Y. Idrissi A. K. El Khamlichi B. El Fallah-Seghrouchni A. (2023). Refined edge detection with cascaded and high-resolution convolutional network. Pattern Recognit. 138:109361. 10.1016/j.patcog.2023.109361

[B12] Ghandorh H. Boulila W. Masood S. Koubaa A. Ahmed F. Ahmad J. (2022). Semantic segmentation and edge detection approach to road detection in very high resolution satellite images. Remote Sens. 14:613. 10.3390/rs14030613

[B13] Guo F. Zhang G. Zhang Q. Zhao R. Deng M. Xu K. (2018). Speckle suppression by weighted euclidean distance anisotropic diffusion. Remote Sens. 10:722. 10.3390/rs10050722

[B14] Guobin C. Sun Z. Zhang L. (2020). Road identification algorithm for remote sensing images based on wavelet transform and recursive operator. IEEE Access 8, 141824–141837. 10.1109/ACCESS.2020.3012997

[B15] Gupta B. Lamba S. S. (2021). An efficient anisotropic diffusion model for image denoising with edge preservation. Comp. Mathem. Appl. 93, 106–119. 10.1016/j.camwa.2021.03.029

[B16] He J. Zhang S. Yang M. Shan Y. Huang T. (2022). Bdcn: Bi-directional cascade network for perceptual edge detection. IEEE Trans. Pattern Anal. Mach. Intell. 44, 100–113. 10.1109/TPAMI.2020.3007074 32750803

[B17] He K. Zhang X. Ren S. Sun J. (2016). “Deep residual learning for image recognition,” in Proceedings of the IEEE Conference on Computer Vision and Pattern Recognition (Las Vegas, NV: IEEE), 770–778. 10.1109/CVPR.2016.90

[B18] Jing J. Gao T. Zhang W. Gao Y. Sun C. (2022a). Image feature information extraction for interest point detection: a comprehensive review. IEEE Trans. Pattern Anal. Mach. Intell. 45, 4694–4712. 10.1109/TPAMI.2022.3201185 36001516

[B19] Jing J. Liu S. Wang G. Zhang W. Sun C. (2022b). Recent advances on image edge detection: a comprehensive review. Neurocomputing 503, 259–271. 10.1016/j.neucom.2022.06.083

[B20] Kelm A. P. Rao V. S. Zölzer U. (2019). “Object contour and edge detection with refinecontournet,” in Computer Analysis of Images and Patterns (Cham: Springer), 246–258.

[B21] Le T. Duan Y. (2020). REDN: a recursive encoder-decoder network for edge detection. IEEE Access 8, 90153–90164. 10.1109/ACCESS.2020.2994160 32542175 PMC7295132

[B22] Lei T. Song W. Zhang W. Du X. Li C. He L.. (2024). Semi-supervised 3d medical image segmentation using multi-consistency learning with fuzzy perception-guided target selection. IEEE Trans. Radiat. Plasma Med. Sci. 421–432. 10.1109/TRPMS.2024.3473929

[B23] Li L. Zhou Z. Wu S. Cao Y. (2023). Multi-scale edge-guided learning for 3d reconstruction. ACM Trans. Multimedia Comp. Commun. Appl. 19, 1–24. 10.1145/3568678

[B24] Li Y. Bi Y. Zhang W. Sun C. (2019). Multi-scale anisotropic gaussian kernels for image edge detection. IEEE Access 8, 1803–1812. 10.1109/ACCESS.2019.2962520

[B25] Liao Y. Gao Y. Zhang W. (2025). Dynamic accumulated attention map for interpreting evolution of decision-making in vision transformer. Pattern Recognit. 2025:111607. 10.1016/j.patcog.2025.111607

[B26] Liu H. Yang Z. Zhang H. Wang C. (2022). Edge detection with attention: from global view to local focus. Pattern Recognit. Lett. 154:99–109. 10.1016/j.patrec.2022.01.006

[B27] Liu Y. Cheng M.-M. Hu X. Bian J.-W. Zhang L. Bai X.. (2019). Richer convolutional features for edge detection. IEEE Trans. Pattern Anal. Mach. Intell. 41, 1939–1946. 10.1109/TPAMI.2018.2878849 30387723

[B28] Liu Y. Cheng M.-M. Hu X. Wang K. Bai X. (2017). “Richer convolutional features for edge detection,” in Proceedings of the IEEE Conference on Computer Vision and Pattern Recognition (Cham: IEEE), 3000–3009.

[B29] Ma P. Yuan H. Chen Y. Chen H. Weng G. Liu Y. (2024). A laplace operator-based active contour model with improved image edge detection performance. Digit. Signal Process. 151:104550. 10.1016/j.dsp.2024.104550

[B30] Ma W. Gong C. Xu S. Zhang X. (2020). Multi-scale spatial context-based semantic edge detection. Inform. Fusion 64, 238–251. 10.1016/j.inffus.2020.08.014

[B31] Mély D. A. Kim J. McGill M. Guo Y. Serre T. (2016). A systematic comparison between visual cues for boundary detection. Vision Res. 120, 93–107. 10.1016/j.visres.2015.11.007 26748113

[B32] Park Y.-H. Seo J. Moon J. (2020). CafeNet: class-agnostic few-shot edge detection network. arXiv [preprint] arXiv:2003.08235. 10.48550/arXiv.2003.08235

[B33] Pu M. Huang Y. Liu Y. Guan Q. Ling H. (2022). “Edter: edge detection with transformer,” in Proceedings of the IEEE Conference on Computer Vision and Pattern Recognition (New Orleans, LA: IEEE), 1402–1412. 10.1109/CVPR52688.2022.00146

[B34] Qiu B. Guo J. Kraeima J. Glas H. H. Zhang W. Borra R. J.. (2021). Recurrent convolutional neural networks for 3D mandible segmentation in computed tomography. J. Pers. Med. 11:492. 10.3390/jpm11060492 34072714 PMC8229770

[B35] Ren J. Li C. An Y. Zhang W. Sun C. (2024). Few-shot fine-grained image classification: a comprehensive review. AI 5, 405–425. 10.3390/ai5010020

[B36] Romani L. Rossini M. Schenone D. (2019). Edge detection methods based on rbf interpolation. J. Comput. Appl. Math. 349, 532–547. 10.1016/j.cam.2018.08.006

[B37] Shui P.-L. Zhang W.-C. (2012). Noise-robust edge detector combining isotropic and anisotropic gaussian kernels. Pattern Recognit. 45, 806–820. 10.1016/j.patcog.2011.07.020

[B38] Silberman N. Hoiem D. Kohli P. Fergus R. (2012). “Indoor segmentation and support inference from RGBD images,” in Proceedings of the European Conference on Computer Vision (Cham: Springer), 746–760.

[B39] Simonyan K. Zisserman A. (2014). Very deep convolutional networks for large-scale image recognition. arXiv [preprint] arXiv:1409.1556. 10.48550/arXiv.1409.1556

[B40] Soria X. Sappa A. Humanante P. Akbarinia A. (2023). Dense extreme inception network for edge detection. Pattern Recognit. 139:109461. 10.1016/j.patcog.2023.109461

[B41] Su Z. Liu W. Yu Z. Hu D. Liao Q. Tian Q.. (2021). “Pixel difference networks for efficient edge detection,” in Proceedings of the IEEE Conference on Computer Vision and Pattern Recognition (Montreal, QC,: IEEE), 5117–5127.

[B42] Wang J. Lu J. Yang J. Wang M. Zhang W. (2024). An unbiased feature estimation network for few-shot fine-grained image classification. Sensors 24:7737. 10.3390/s24237737 39686274 PMC11644890

[B43] Wang Y. Zhao X. Huang K. (2017). “Deep crisp boundaries,” in Proceedings of the IEEE Conference on Computer Vision and Pattern Recognition (Honolulu, HI: IEEE), 3892–3900.

[B44] Xian R. Xiong X. Peng H. Wang J. de Arellano Marrero A. R. Yang Q. (2024). Feature fusion method based on spiking neural convolutional network for edge detection. Pattern Recognit. 147:110112. 10.1016/j.patcog.2023.110112

[B45] Xie S. Tu Z. (2017). Holistically-nested edge detection. Int. J. Comput. Vis. 125, 3–18. 10.1007/s11263-017-1004-z

[B46] Xuan W. Huang S. Liu J. Du B. (2022). Fcl-net: Towards accurate edge detection via fine-scale corrective learning. Neural Netw. 145, 248–259. 10.1016/j.neunet.2021.10.022 34773900

[B47] Ye Y. Xu K. Huang Y. Yi R. Cai Z. (2024). “Diffusionedge: diffusion probabilistic model for crisp edge detection,” in Proceedings of the AAAI Conference on Artificial Intelligence (Cham: AAAI), 6675–6683.

[B48] You N. Han L. Zhu D. Song W. (2023). Research on image denoising in edge detection based on wavelet transform. Appl. Sci. 13:1837. 10.3390/app13031837

[B49] Zhang J. Shao Z. Ding Q. Huang X. Wang Y. Zhou X.. (2023). AERNet: an attention-guided edge refinement network and a dataset for remote sensing building change detection. IEEE Trans. Geosci. Remote Sens. 17:3300533. 10.1109/TGRS.2023.3300533

[B50] Zhang W. Sun C. (2019). Corner detection using second-order generalized gaussian directional derivative representations. IEEE Trans. Pattern Anal. Mach. Intell. 43, 1213–1224. 10.1109/TPAMI.2019.2949302 31670662

[B51] Zhang W. Sun C. (2020). Corner detection using multi-directional structure tensor with multiple scales. Int. J. Comput. Vis. 128, 438–459. 10.1007/s11263-019-01257-2

[B52] Zhang W. Sun C. Breckon T. Alshammari N. (2019). Discrete curvature representations for noise robust image corner detection. IEEE Trans. Image Proc. 28, 4444–4459. 10.1109/TIP.2019.2910655 30998469

[B53] Zhang W. Sun C. Gao Y. (2023). Image intensity variation information for interest point detection. IEEE Trans. Pattern Anal. Mach. Intell. 45, 9883–9894. 10.1109/TPAMI.2023.3240129 37022077

[B54] Zhang W. Zhao Y. Breckon T. P. Chen L. (2017). Noise robust image edge detection based upon the automatic anisotropic gaussian kernels. Pattern Recognit. 63, 193–205. 10.1016/j.patcog.2016.10.008

[B55] Zhang W. Zhao Y. Gao Y. Sun C. (2024). Re-abstraction and perturbing support pair network for few-shot fine-grained image classification. Pattern Recognit. 148:110158. 10.1016/j.patcog.2023.110158

[B56] Zhang W.-C. Shui P.-L. (2015). Contour-based corner detection via angle difference of principal directions of anisotropic gaussian directional derivatives. Pattern Recognit. 48, 2785–2797. 10.1016/j.patcog.2015.03.021

[B57] Zhang W.-C. Wang F.-P. Zhu L. Zhou Z.-F. (2014). Corner detection using gabor filters. IET Image Process. 8, 639–646. 10.1049/iet-ipr.2013.0641

[B58] Zhang Z. Deng C. Jin W. Peng L. (2022). “Edge detection algorithm based on threshold function de-noising and wavelet neural network,” in Applied Mathematics, Modeling and Computer Simulation (Amsterdam: IOS Press).

[B59] Zheng B. Ji P. Shi Y. Yu Y. (2023). Edge detection ghost imaging using frequency-domain filtering. J. Phys. 2478:122063. 10.1088/1742-6596/2478/12/122063

